# Mini-FLOTAC, an Innovative Direct Diagnostic Technique for Intestinal Parasitic Infections: Experience from the Field

**DOI:** 10.1371/journal.pntd.0002344

**Published:** 2013-08-01

**Authors:** Beatrice Divina Barda, Laura Rinaldi, Davide Ianniello, Henry Zepherine, Fulvio Salvo, Tsetan Sadutshang, Giuseppe Cringoli, Massimo Clementi, Marco Albonico

**Affiliations:** 1 Laboratory of Microbiology San Raffaele Hospital, Milan, Italy; 2 Section of Veterinary Parasitology and Parasitic Diseases, University of Naples Federico II, Naples, Italy; 3 Bukumbi Hospital, Mwanza, Tanzania; 4 Emerging Pathogens Unit, San Raffaele Scientific Institute, Milan, Italy; 5 Tibetan Delek Hospital, Dharamsala, Himachal Pradesh, India; 6 Ivo de Carneri Foundation, Milan, Italy; Swiss Tropical and Public Health Institute, Switzerland

## Abstract

**Background:**

Soil-transmitted helminths and intestinal protozoa infection are widespread in developing countries, yet an accurate diagnosis is rarely performed. The aim of this study was to evaluate the recently developed mini–FLOTAC method and to compare with currently more widely used techniques for the diagnosis of intestinal parasitic infections in different settings.

**Methodology/Principal Findings:**

The study was carried out in Dharamsala, Himachal Pradesh, India, and in Bukumbi, Tanzania. A total of 180 pupils from two primary schools had their stool analyzed (n = 80 in Dharamsala and n = 100 in Bukumbi) for intestinal parasitic infections with three diagnostic methods: direct fecal smear, formol-ether concentration method (FECM) and mini-FLOTAC. Overall, 72% of the pupils were positive for any intestinal parasitic infection, 24% carried dual infections and 11% three infections or more. The most frequently encountered intestinal parasites were *Entamoeba coli*, *Entamoeba histolytica/dispar*, *Giardia intestinalis*, hookworm, (and *Schistosoma mansoni*, in Tanzania). Statistically significant differences were found in the detection of parasitic infections among the three methods: mini-FLOTAC was the most sensitive method for helminth infections (90% mini-FLOTAC, 60% FECM, and 30% direct fecal smear), whereas FECM was most sensitive for intestinal protozoa infections (88% FECM, 70% direct fecal smear, and 68% mini-FLOTAC).

**Conclusion/Significance:**

We present the first experiences with the mini-FLOTAC for the diagnosis of intestinal helminths and protozoa. Our results suggest that it is a valid, sensitive and potentially low-cost alternative technique that could be used in resource-limited settings — particularly for helminth diagnosis.

## Author Summary

The mini-FLOTAC has been recently developed as a novel direct method for the diagnosis of intestinal parasitic infections. Mini-FLOTAC attempts to address the challenge of using modern technology matched with high sensitivity, affordability, and appropriateness of diagnosis in resource-limited settings where intestinal parasitic infections are widespread. We compared accuracy and feasibility of mini-FLOTAC with currently more widely used diagnostic methods, such as the direct fecal smear and the formol-ether concentration method. Our study was carried out in Dharamsala, India, and in Bukumbi, Tanzania in order to evaluate the methods under different field conditions and diverse parasitic infection profiles. Among 180 primary schoolchildren examined, 72% were positive for any intestinal parasitic infection. Mini-FLOTAC detected the highest number of helminth infections (90% sensitivity), whereas the formol-ether concentration was the most sensitive approach for intestinal protozoa infections (88% sensitivity). The logistic advantages of mini-FLOTAC are that the procedure does not require any centrifugation step or expensive equipment, it can be performed on fresh and fixed stool samples, and only requires 10–12 min of preparation before microscopic analysis. Our data suggest that mini-FLOTAC is a promising diagnostic tool for helminth diagnosis; therefore, follow-up studies in other settings are warranted.

## Introduction

Soil-transmitted helminths (STH) (*Ascaris lumbricoides*, *Trichuris trichiura*, hookworm, *Strongyloides stercoralis*) and intestinal protozoa (e.g. *Giardia intestinalis*, *Entamoeba histolytica/dispar*) are widespread in developing countries, especially where poor hygienic conditions facilitate infection with eggs, larvae and cysts through contact with contaminated soil, food or water. Soil-transmitted helminths affect more than 1 billion people worldwide and are part of the neglected tropical diseases (NTDs), which are linked to poverty and underdevelopment [1]. Soil-transmitted helminth infections in endemic countries are a major cause of malnutrition, anemia and growth delay. Often they are linked to minor symptoms such as sub-acute diarrhea or can occur in a subtle and asymptomatic way and are often underreported [2]. Intestinal protozoa infections (especially *G.intestinalis* and *Entamoeba* spp.) are of considerable public health importance [3], [4]. For example, *Giardia* prevalence is 2–7% in developed countries, whereas it is 20–30% in developing countries due to water and food contamination [5]. Around 200 million people are infected around the world with 50,000 new cases occurring every year. *E. histolytica* infects hundreds of millions of people per year; while most individuals are asymptomatic, perpetuating the natural cycle of the organism through fecal excretion of infective cysts, a minority suffers from the severe morbidity associated with invasive disease (approximately 50 million) with an estimated 100,000 dying every year from severe and invasive amebiasis [6].

The technique recommended for the qualitative diagnosis of intestinal parasites (both helminths and intestinal protozoa) is the formol-ether concentration method (FECM) [7], [8], [9] performed on three different samples, but the direct fecal smear on a single sample is used as diagnostic method more often in resource-constrained settings. The recommended quantitative technique for the diagnosis of soil-transmitted helminths is the Kato-Katz method, except for *S. stercoralis*, which requires the Bearmann, the Koga agar plate or the Harada Mori method as recommended direct diagnostic techniques [8].

Recently, fecal egg count (FEC) techniques used for the diagnosis of helminths in veterinary parasitology, such as the FLOTAC [10], [11] and the McMaster [12] techniques have been successfully used for diagnosis of soil-transmitted helminths infections in humans [13], [14], [15], [16].

Molecular tools such as polymerase chain reaction (PCR) and multiplex PCR are now increasingly common as diagnostic methods for parasitic infection in laboratories where the equipment is available, although this is often not the case in rural and district laboratories in developing countries [17], [18], [19], [20].

Despite their high sensitivity, a main limitation of the FLOTAC techniques is the complexity of the method which involve centrifugation of the sample with a specific device, equipment that is often not available in laboratories in developing countries [14]. To overcome this bottleneck, under the “FLOTAC strategy” of improving the quality of copromicroscopic diagnosis, a new simplified device has been developed, namely the mini-FLOTAC. One of the main advantages of this new method is that it can be more easily transferred and carried out in laboratories with limited facilities due to the lack of a centrifugation step.

The aim of this study was to evaluate and compare widely used, standard qualitative diagnostic techniques with the mini-FLOTAC in different settings. The current report focuses on the diagnostic accuracy and feasibility of the different methods, placing particular emphasis on the application of the mini-FLOTAC method in rural/peripheral laboratories with basic facilities.

## Materials and Methods

### Ethic Statement

The overall protocol of the study on the evaluation of the mini-FLOTAC was reviewed and approved by the Ethics Committee of the Faculty of Medicine, San Raffaele Hospital, Milan, Italy. For each proposed study site a separate ethical clearance was obtained from the local review board.

All children were given an informed consent form to be read and approved by their parents/guardian before being enrolled into the study. All children diagnosed positive for intestinal parasitic infections by any of the methods performed were treated according to standard protocol in use within the country. Data were kept anonymous and patients were identified by code; the study data were safely filed and stored in a cabinet within the data management unit of each research site and remained confidential.

### Study Sites

The study sites have been chosen among places where hospitals and health facilities are currently collaborating with an Italian non-governmental organization (Italian Association for Solidarity among People) and San Raffaele hospital. In particular, collaboration focused on facilities that were in need of technical support with laboratory diagnosis for intestinal parasitic infections.

The first part of the study was carried out in February and March 2012 in a school in Dharamsala, Himachal Pradesh, India. Available data from Tibetan Delek hospital show that the prevalence of intestinal parasitic infection is around 10% in patients seen in the outpatient department, but this number is likely to be underestimated as stool diagnosis is made by direct smear. Published data show that *Giardia* infection is estimated to be around 20%, and the most common infection among soil-transmitted helminths is *A. lumbricoides* (11%) [21], [22]. All pupils (n = 80) of a school under the authority of the Department of Education of the Central Tibetan Administration in exile were analyzed for intestinal parasites.

The second part of the study was conducted in May and June 2012 in Bukumbi, Mwanza district, Tanzania. In this region the commonest intestinal parasites are hookworm, *Schistosoma mansoni*, *S. stercoralis*, *G. intestinalis* and *Entoamoeba* spp. as determined from hospital records based on direct fecal smear. One hundred (n = 100) children were randomly selected from the only primary school in Bukumbi.

### Sample Size and Randomization

The sample size has been calculated on the basis of mainly historical and unpublished data on the prevalence of intestinal parasitic infections in the study areas, conservatively estimated to be around 20%. In order to have an ideal number of positive/negatives (50%) to determine comparison among the techniques, the adequate sample size to have a significant difference with 95% confidence interval (CI) and with 80% of power was 88 in each site.

The selection between the two primary schools in Dharamsala was made at random and all 80 children were examined in the Tibetan school selected. Three classes of children (grades 2, 3 and 4) from the only primary school in Bukumbi were selected at random and all children in those classes were examined for intestinal parasites.

### Parasitological Methods

Stool containers were distributed to the children together with the consent forms, and the next day one fecal sample (minimum 12 g) was collected from each child and analysed on the same day. Samples were examined in parallel by direct smear, FECM and mini-FLOTAC in the hospital laboratory, and were processed and blindly read by two experienced parasitologists (BB and DI among the authors).

In brief, approximately 2 mg of stool were used to perform a direct fecal smear [7].

With regard to the mini-FLOTAC, the technique evolved from FLOTAC techniques [10], [11], adapted in order to perform the techniques without the necessity of a centrifugation step. The mini-FLOTAC comprises two physical components, the base and the reading disc. There are two 1-ml flotation chambers, which are designed for optimal examination of fecal sample suspensions in each flotation chamber (total volume = 2 ml) and which permits a maximum magnification of 400×.

**Figure 1:**
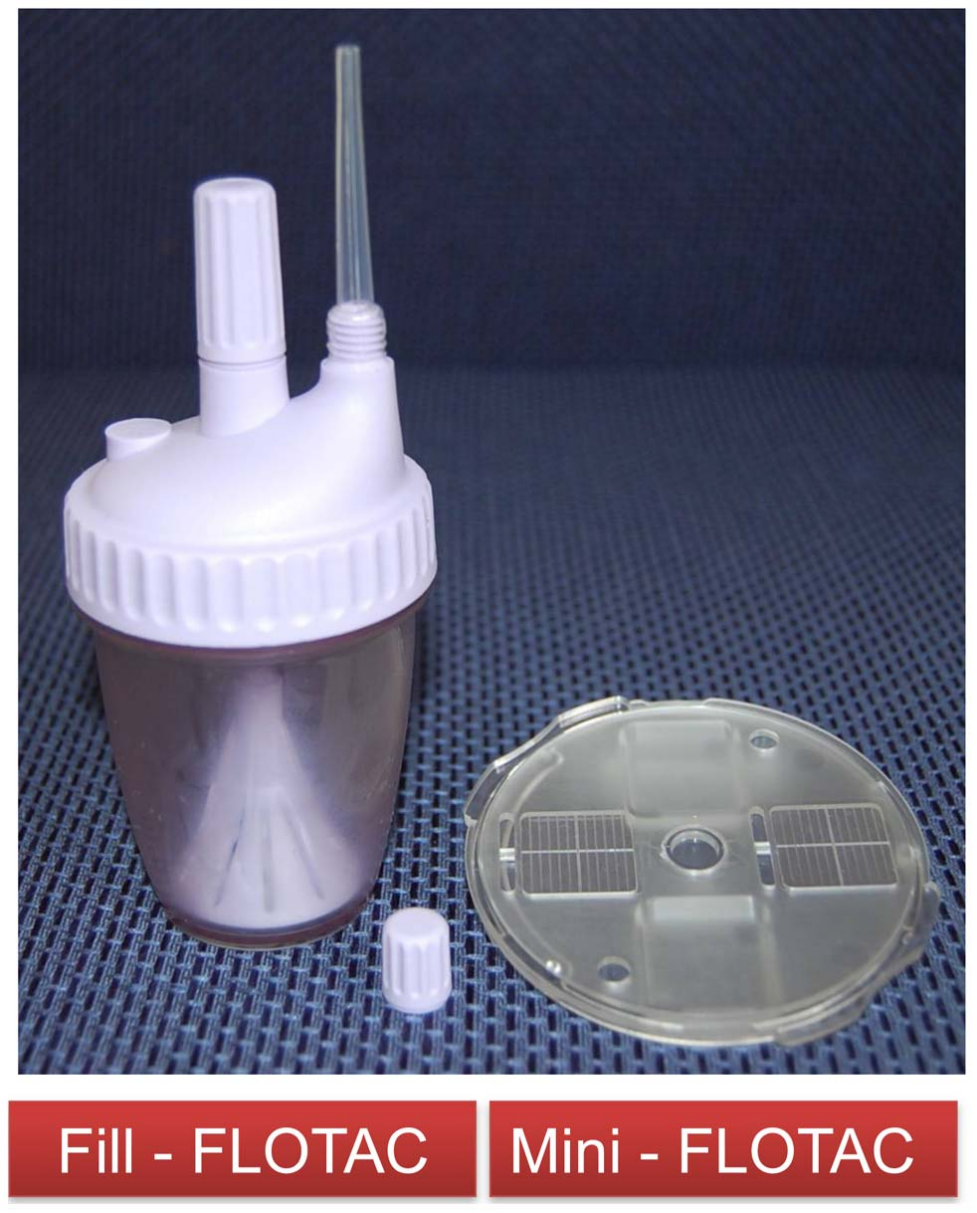
The fill-FLOTAC and the mini-FLOTAC kit.

**Figure 2:**
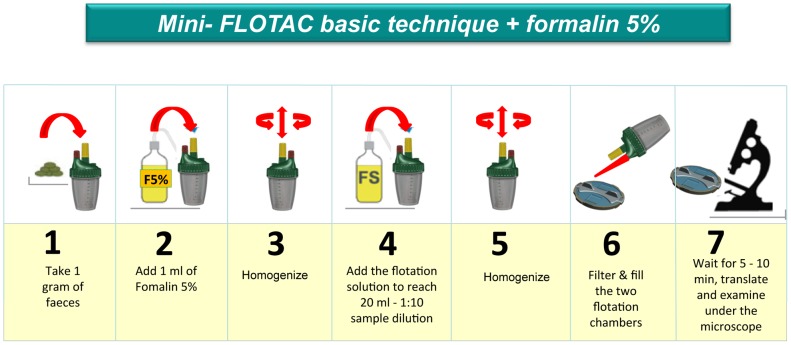
The steps of the mini-FLOTAC technique.

Fill-FLOTAC are disposable sampling devices, which are part of the FLOTAC and mini-FLOTAC kits [10], [11]. They consist of a container, a collector and a filter (Figure 1). These kits facilitate the performance of the first four consecutive steps of the mini-FLOTAC techniques, i.e. collection (including weighing), homogenization, filtration and filling. The process of the mini-FLOTAC is illustrated in Figure 2.

The stools were processed as follows for the mini-FLOTAC basic technique (analytic sensitivity = 10 eggs or cysts per gram of feces). Eight grams of stool were placed in the fill-FLOTAC, diluted with 8 ml of formalin 5%, and thoroughly homogenized and filtered. Two ml of the suspension (1 g of stool+1 ml of formalin) were directly added to 18 ml of each of the two floatation solutions (FS), namely FS2 (saturated sodium chloride; specific gravity (s.g.) = 1.20) and FS7 (zinc sulphate; s.g. = 1.35). The flotation solutions are the same described in the FLOTAC protocols. The FS2 solution is recommended for the diagnosis of soil-transmitted helminths, the FS7 solution is recommended for *S. mansoni* and for intestinal protozoa [10]. Two mini-FLOTAC were performed for each sample, one filled with the fecal suspension in FS2 and the other with the fecal suspension in FS7. Before reading the slide and translating the reading dish, an average time of 10 min was needed for the eggs and cysts to float.

Two ml of the initial 1∶1 solution (1 g of faeces plus 1 ml of 5% formalin solution) in the fill-FLOTAC were used to perform the FECM according to WHO recommendations [7].

Eggs of STHs were detected and counted. In addition, parasitic elements of other helminth genera (e.g. *Strongyloides*, *Enterobius*, *Hymenolepis*, *Taenia*) and intestinal protozoa were detected. The comparison between the three techniques was made on qualitative diagnosis as direct smear and FECM are not quantitative methods.

### Statistical Analysis

Results were entered in an Excel file. Analysis was performed using the EpiData program (version 3.1; Area of Health Analysis and Information Systems Pan American Health Organization (PAHO/WHO) January 2006). The results were analysed by 2×2 contingency tables and Cohen's kappa statistics was calculated to assess the agreement among all the three diagnostic techniques. Kappa (k) statistic was employed to determine to strength of agreement using follows criteria: ≤0 = poor, 0.01–0.20 = slight, 0.21–0.40 = fair, 0.41–0.60 = moderate, 0.61–0.80 = substantial and 0.81–1 = almost perfect. We checked for any significant difference by calculating interference about proportions (p) in two independent populations with the level of significance set at p value<0.05, and a 95% CI was calculated. Sensitivity (probability to detect a “true” positive case) and negative predictive value (NPV; probability for a negative result to be a “true” negative case) were calculated for each method. The total number of positive samples detected by any of the methods was taken as diagnostic “gold” standard for each parasite species.

## Results

Out of 200 students that were enrolled, 180, 47% (85/180) girls and 53% (95/180) boys, aged between 6 and 18 years (mean age 12 years) returned the sample and had their stool analysed for intestinal parasites.

**Figure 3:**
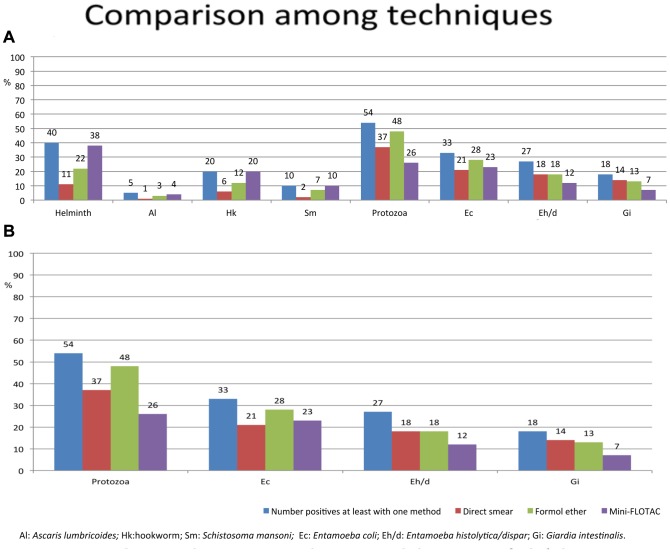
A) Prevalence of intestinal helminth and protozoa infections detected by each of the three methods. B) Prevalence focus on protozoa, Entamoeba coli, Entamoeba histolytica/dispar and Giardia intestinalis results.

The results of the study are shown in Table 1, Table 2, and in Figure 3. Overall, 72% (129/180) of the samples were positive for any intestinal parasitic infection, 24% (43/180) carried double infections, and 11% (20/180) three infections or more.

**Table 1:** Sensitivity and negative predictive value (NPV) of each diagnostic method.

	Tot (n = 180)	Direct smear			FECM			Mini-FLOTAC FS2			Mini-FLOTAC FS7		
	N. positives	N. positives	sensitivity	NPV	N. positives	sensitivity	NPV	N. positives	sensitivity	NPV	N. positives	sensitivity	NPV
**Helminth**	72	19	0.27	0.68	40	0.56	0.78	45	0.63	0.81	67	0.94	0.96
**Hookworm**	36	10	0.27	0.84	22	0.60	0.90	36	0.97	0.99	33	0.89	0.97
***S. mansoni***	22	4	0.18	0.89	12	0.36	0.92	0	N/A	N/A	18	0.82	0.98
**Protozoa**	97	66	0.70	0.75	86	0.88	0.88	0	N/A	N/A	64	0.68	0.74
***E. coli***	59	37	0.63	0.84	51	0.87	0.94	0	N/A	N/A	42	0.71	0.88
***E. histolytica*** **/** ***dispar***	48	32	0.67	0.89	33	0.68	0.89	0	N/A	N/A	22	0.46	0.84
***G. intestinalis***	33	26	0.82	0.95	23	0.70	0.94	0	N/A	N/A	12	0.40	0.87

**Table 2:** Qualitative diagnosis of intestinal helminth and protozoan infections by direct smear, FECM and mini-FLOTAC method.

	Combined results		Direct smear	FECM	mini - FLOTAC	
	N pos (%)	K Cohen (CI 95%)^1^	N pos (%)	N pos (%)	N pos (%)	K Cohen(CL 95%)^2^
**Helminth**	72 (40)	0.41 (0.29–0.52)	19 (11)	40 (22)	68 (38)	0.54 (0.41–0.66)
Hookworm	36 (20)	0.53 (0.39–0.67)	10 (6)	22 (12)	36 (20)	0.72 (0.58–0.85)
*S. mansoni*	18 (10)	0.40 (0.19–0.61)	4 (2)	12 (7)	18 (10)	0.49 (0.26–0.72)
**Protozoa**	97 (54)	0.57 (0.48–0.67)	66 (37)	86 (48)	64 (36)	0.70 (0.60–0.80)
*E. coli*	59 (33)	0.64 (0.55–0.74)	37 (21)	51 (28)	42 (23)	0.81 (0.71–0.90)
*E. histolytica*/*dispar*	48 (27)	0.48 (0.35–0.60)	32 (18)	33 (18)	22 (12)	0.60 (0.43–0.76)
*G. intestinalis*	33 (18)	0.57 (0.42–0.73)	26 (14)	23 (13)	12 (7)	0.66 (0.47–0.84)

1 K Cohen among all methods.

2 K Cohen between two methods.

We found a difference in helminth distribution according to the geographical area: hookworm (20%), S. *mansoni* (10%), *S. stercoralis* and *E. vermicularis* (2%) were found in the African school, whereas *A. lumbricoides* (5%), *T. trichiura* (2%), *H. nana* and *Taenia* spp. (1%) were found in the Indian school. Intestinal protozoa were more homogeneously found in the two study sites.

Mini-FLOTAC detected a higher number of helminth infections than the FECM or direct smear (38%, 22% and 11%, respectively) and the differences were significant (p<0.002 and p<0.001, respectively). Mini-FLOTAC had a sensitivity of around 90%, followed by FECM and direct fecal smear (60% and 30% respectively). The FECM detected the highest number of intestinal protozoa infection followed by the direct fecal smear and mini-FLOTAC technique (48%, 37% and 36%, respectively). The differences between FECM and mini-FLOTAC/direct fecal smear were significant (p<0.04 and p<0.02, respectively). Results are shown in Figure 3. The most sensitive method for intestinal protozoa diagnosis was FECM (88%), followed by direct fecal smear and mini-FLOTAC (70% and 68% respectively). The NPV were above 70% with all the three methods for both helminth and intestinal protozoa and they did not differ significantly (Table 1).

The agreement between techniques is shown in Table 2. The agreement among the three techniques was only moderate (k = 0.40; p<0.001) and the best match was between FECM and mini-FLOTAC (k = 0.49–0.81; p<0.001). Eighteen children were found positive for other parasitic infections such as *A. lumbricoides*, *E. vermicularis*, *Hymenolepis nana*, *S. stercoralis*, *Taenia* spp. *and T. trichiura*, but they were too few for a meaningful statistic and were not included in the analysis.

Considering the practical feasibility of the techniques, mini-FLOTAC took approximately 12 min to process a sample: 2 min to prepare the sample, 10 min to wait for the eggs/cysts to float, and 5–7 min to read the reading grid. The FECM on average took 2 min to prepare the sample, 10 min of centrifugation and 3 min to read the slide (a total of 15 min). The simplest and quickest method to perform was the direct smear as it did not require any further step beyond approximately 3 min for reading of the slide.

## Discussion

For the first time the mini-FLOTAC apparatus was used for detecting soil-transmitted helminths and other helminths and intestinal protozoa and compared with two diagnostic techniques that are widely used in human parasitology.

In the present study, adhering to standard protocols, all techniques revealed the same intestinal protozoa species (*E. coli*, *E. histolytica/dispar*, *G. intestinalis*), whereas the mini–FLOTAC revealed more helminth species than the two other techniques (*E. vermicularis* was found only with mini–FLOTAC and *T. trichiura* was not detected by the direct fecal smear). The number of individuals identified as positive by each technique varied considerably from one species to another.

Our results show, similar to Levecke et al [23] for FLOTAC, that the mini–FLOTAC turned out to be the most sensitive approach (95% of the positive samples) for helminth diagnosis. An important observation of our study is that the diagnostic accuracy of the mini-FLOTAC changes according to the FS used: hookworm and *E. vermicularis* were more accurately diagnosed by FS2, whilst *S. mansoni* was diagnosed with a higher sensitivity by the FS7 solution, thus supporting the application of the FLOTAC method [11], [24].

When considering intestinal protozoa infections, the diagnostic pattern changes, and our results demonstrated relatively poor performance of mini-FLOTAC. These data differ substantially from the ones reported by Becker et al. [13], where FLOTAC was found to be more sensitive for *E. coli* and for *G. intestinalis* detection, whilst FECM was more sensitive for *E.histolytica/dispar*. It must be considered however, that the centrifugation step makes the FLOTAC different and not easily comparable to the mini-FLOTAC, especially for the diagnosis of intestinal protozoa. In the present papers we did not compare the FLOTAC with the mini-FLOTAC as the latter is not a replacement of the former, but the two methods should be used in different laboratory settings according to the facilities and equipment available. We realised, however, that a standard direct comparison of the mini-FLOTAC versus the FLOTAC method could be important both to compare the sensitivity of the methods and to evaluate their feasibility and cost-benefits.

For the diagnosis of the protozoa in area with heavy parasite load and limited resources, the direct smear could be considered a sufficient method, as in this study it does not lose too much sensitivity compared to the FECM. It is worth noticing that in our study the sensitivity of the direct fecal smear for the diagnosis of *G.intestinalis* was even greater than the FECM, which could be explained by the fact that most of the samples were positive for trophozoites that were killed by formol-preservation, and therefore resulted negative with the other two techniques.

Our study suffers from some limitations, which could partially explain the discordance between the three methods in parasitic diagnosis. The agreement between the three methods was variable (k values ranged between 0.4 and 0.8), but generally moderate (k = 0.6). Low agreement in protozoa diagnosis has been observed in other studies in which the same set of samples was analysed in different referral laboratories [25]. Due to low prevalence of other helminth infections, the findings of this study are mainly based on hookworm and *S. mansoni* diagnosis and further studies are needed in different settings to formulate more reliable and conclusive recommendations for use of mini-FLOTAC for other soil-transmitted helminths diagnosis.

It is important to consider that the mini- FLOTAC technique is still under refinement and two main aspects need to be addressed in more details, especially for intestinal protozoa diagnosis. Firstly, stool consistency plays an important role, especially in light infections, as loose stools contain a lower amount of cysts. Mucous stool could be treated adding a mucosolvant or, where the laboratory facilities can afford it, a centrifugation step with ether could be performed in order to eliminate the debris. The visibility of internal structures is often impaired because of debris, thus rendering complicated the differentiation among *Entamoeba* spp. Moreover, the reading at high power of magnitude (400×) was not clear as the reading disk of the mini-FLOTAC does not yet allow perfect visibility.

Secondly, mini–FLOTAC has been tested only using two different flotation solutions (saturated NaCl and Zinc sulphate), but other FS could be tested and the visibility of the samples compared. Furthermore, the flotation solution interacts with and may alter the external membrane of parasites (such as *E. coli*, *G. intestinalis*, *S. mansoni*) somehow distorting their images from the classical pattern. For this reason, well-trained laboratory technicians are needed for reading mini-FLOTAC and a teaching Atlas that could help reading the sample might be a useful tool to add to the mini-FLOTAC kit.

An advantage of the mini-FLOTAC technique is that it permits work with fixed fecal sample, but can also be performed on fresh samples. This allows the possibility of examining the samples in different days and also improves the quality control process; in addition, the combined use of the fill-FLOTAC device prevents any hazard of contamination of the operator.

A series of studies is further warranted to compare the quantitative performance of the mini –FLOTAC with the other recommended standard techniques for helminth diagnosis such as the Kato Katz and the McMaster. This is an important aspect in helminth control as intensity of infections measured as eggs per gram is directly related to morbidity, and is an important indicator to monitor the impact of control programs as well as drug efficacy [23], [26]. The preliminary qualitative results of the mini-FLOTAC performance from this study should foster the planning of a robust independent multicentric trial that compares the mini-FLOTAC with standard protocols both on qualitative and quantitative diagnosis of helminth infections.

Considering the cost-benefit and the feasibility of the techniques, we demonstrated that mini-FLOTAC takes approximately the same time to process the sample as the FECM. As for the cost of the equipment, the only cost for the mini-FLOTAC (apart the cost of the kit which is presently given free of charge for research purposes) is the purchase of sodium chloride (NaCl) and zinc sulphate (ZnSO_4_) for the flotation solutions, whereas the FECM requires a centrifuge, formol and ether that are not always easy to purchase, especially in laboratories with limited resources. All mini-FLOTAC reading disks and the fill-FLOTAC containers are reusable after careful washing. However, the fact that zinc sulphate is a quite expensive salt and not easily available in peripheral laboratories should be taken into consideration.

To conclude, this study demonstrates that mini-FLOTAC is definitely a sensitive and relatively simple technique for the qualitative diagnosis of helminth infections, whilst it still could be improved for the diagnosis of intestinal protozoa. We believe that this work is a step forward in the fight against NTDs, because neglected diagnostics sustain the vicious cycle of hidden diseases and poor access to clinical management and treatment. Innovative direct diagnostic tools that match modern technology and sensitivity with affordability and feasibility in resource-limited settings are most needed, and the development of the mini-FLOTAC kit is an advance towards this direction.

## Supporting Information

Figure S1 **STARD checklist.**
(DOC)

Figure S2 **Flow diagram.**
(DOCX)
