# Supplementary material for: Mini-FLOTAC, an Innovative Direct Diagnostic Technique for Intestinal Parasitic Infections: Experience from the Field
Source: PLoS Negl Trop Dis. 2013 Aug 1;7(8):e2344. doi: 10.1371/journal.pntd.0002344 (PMC3731229; doi:10.1371/journal.pntd.0002344)
Supplement: Figure S1 — STARD checklist. (DOC) [file pntd.0002344.s001.doc]

# STARD checklist for reporting of studies of diagnostic accuracy

*(version January 2003)*

| **Section and Topic** | **Item**  **#** |  | **On page #** |
| --- | --- | --- | --- |
| TITLE/ABSTRACT/  KEYWORDS | 1 | Identify the article as a study of diagnostic accuracy (MeSH heading 'sensitivity and negative predictive value'). | 1 |
| INTRODUCTION | 2 | State the research questions or study aims, such as comparing accuracy between tests. | 5 |
| METHODS |  |  |  |
| *Participants* | 3 | The study population: The inclusion and exclusion criteria, setting and locations where data were collected. | 6 |
|  | 4 | Participant recruitment: recruitment was based on the fact that the participants had received the index tests or the reference standard | 7 |
|  | 5 | Participant sampling: the study population was a consecutive series of participants defined by the selection criteria in item 3 and 4. | 7 |
|  | 6 | Data collection: data collection was planned before the index test and reference standard were performed (prospective study) | 7 |
| *Test methods* | 7 | The reference standard and its rationale. | 7-8 |
|  | 8 | Technical specifications of material and methods involved including how and when measurements were taken, and/or cite references for index tests and reference standard. | 7-8 |
|  | 9 | Definition of and rationale for the units, cut-offs and/or categories of the results of the index tests and the reference standard. | 8 |
|  | 10 | The number, training and expertise of the persons executing and reading the index tests and the reference standard. | 7 |
|  | 11 | The readers of the index tests and reference standard were blind (masked) to the results of the other test and describe any other clinical information available to the readers. | 7 |
| *Statistical methods* | 12 | Methods for calculating or comparing measures of diagnostic accuracy, and the statistical methods used to quantify uncertainty (e.g. 95% confidence intervals). | 8 |
|  | 13 | Methods for calculating test reproducibility, if done. |  |
| RESULTS |  |  |  |
| *Participants* | 14 | When study was performed, including beginning and end dates of recruitment. | 6 |
|  | 15 | Clinical and demographic characteristics of the study population (at least information on age, gender, spectrum of presenting symptoms). | 9 |
|  | 16 | The number of participants satisfying the criteria for inclusion who did or did not undergo the index tests and/or the reference standard; describe why participants failed to undergo either test (a flow diagram is strongly recommended). |  |
| *Test results* | 17 | Time-interval between the index tests and the reference standard, and any treatment administered in between. | 6-8 |
|  | 18 | Distribution of severity of disease (define criteria) in those with the target condition; other diagnoses in participants without the target condition. |  |
|  | 19 | A cross tabulation of the results of the index tests (including indeterminate and missing results) by the results of the reference standard; for continuous results, the distribution of the test results by the results of the reference standard. | 9, Table 1 and Table 2 |
|  | 20 | Any adverse events from performing the index tests or the reference standard. |  |
| *Estimates* | 21 | Estimates of diagnostic accuracy and measures of statistical uncertainty (e.g. 95% confidence intervals). | 9, Table 1 |
|  | 22 | How indeterminate results, missing data and outliers of the index tests were handled. |  |
|  | 23 | Estimates of variability of diagnostic accuracy between subgroups of participants, readers or centers, if done. |  |
|  | 24 | Estimates of test reproducibility, if done. |  |
| DISCUSSION | 25 | Discuss the clinical applicability of the study findings. | 11-13 |
