# Supplementary material for: Mini-FLOTAC, an Innovative Direct Diagnostic Technique for Intestinal Parasitic Infections: Experience from the Field
Source: PLoS Negl Trop Dis. 2013 Aug 1;7(8):e2344. doi: 10.1371/journal.pntd.0002344 (PMC3731229; doi:10.1371/journal.pntd.0002344)
Supplement: Figure S2 — Flow diagram. (DOCX) [file pntd.0002344.s002.docx]

20 did not return the sample

54 negatives

126 positives

95 negatives

85

positives

131

positives

49 negatives

180 mini-FLOTAC

180 FECM

180 direct smear

200 Eligible patients
